# Self‐adaption and texture generation: A hybrid loss function for low‐dose CT denoising

**DOI:** 10.1002/acm2.14113

**Published:** 2023-08-11

**Authors:** Zhenchuan Wang, Minghui Liu, Xuan Cheng, Jinqi Zhu, Xiaomin Wang, Haigang Gong, Ming Liu, Lifeng Xu

**Affiliations:** ^1^ Yangtze Delta Region Institute(Quzhou), University of Electronic Science and Technology of China Quzhou China; ^2^ The Quzhou Affiliated Hospital of Wenzhou Medical University, Quzhou People's Hospital Quzhou China; ^3^ Tianjin Normal University Tianjin China; ^4^ University of Electronic Science and Technology of China Chengdu China

**Keywords:** CT, deep learning, denoise, hybrid loss

## Abstract

**Background:**

Deep learning has been successfully applied to low‐dose CT (LDCT) denoising. But the training of the model is very dependent on an appropriate loss function. Existing denoising models often use per‐pixel loss, including mean abs error (MAE) and mean square error (MSE). This ignores the difference in denoising difficulty between different regions of the CT images and leads to the loss of large texture information in the generated image.

**Purpose:**

In this paper, we propose a new hybrid loss function that adapts to the noise in different regions of CT images to balance the denoising difficulty and preserve texture details, thus acquiring CT images with high‐quality diagnostic value using LDCT images, providing strong support for condition diagnosis.

**Methods:**

We propose a hybrid loss function consisting of weighted patch loss (WPLoss) and high‐frequency information loss (HFLoss). To enhance the model's denoising ability of the local areas which are difficult to denoise, we improve the MAE to obtain WPLoss. After the generated image and the target image are divided into several patches, the loss weight of each patch is adaptively and dynamically adjusted according to its loss ratio. In addition, considering that texture details are contained in the high‐frequency information of the image, we use HFLoss to calculate the difference between CT images in the high‐frequency information part.

**Results:**

Our hybrid loss function improves the denoising performance of several models in the experiment, and obtains a higher peak signal‐to‐noise ratio (PSNR) and structural similarity index (SSIM). Moreover, through visual inspection of the generated results of the comparison experiment, the proposed hybrid function can effectively suppress noise and retain image details.

**Conclusions:**

We propose a hybrid loss function for LDCT image denoising, which has good interpretation properties and can improve the denoising performance of existing models. And the validation results of multiple models using different datasets show that it has good generalization ability. By using this loss function, high‐quality CT images with low radiation are achieved, which can avoid the hazards caused by radiation and ensure the disease diagnosis for patients.

## 1 INTRODUCTION

X‐ray computed tomography (CT)^1^ is widely used in clinical screening, diagnosis, and intervention because of its fast scanning time and clear image. However, its high radiation puts patients at risk of gene damage and malignant tumor.^2^ Epidemiological studies have shown that even the radiation dose of two or three times CT scans can significantly increase the risk of cancer, especially in children.^3^ Therefore, according to the ALARA principle, low‐dose CT examination is the trend of clinical scanning mode selection in the future. However, when X‐ray flux is reduced,^4^, ^5^ X‐rays cannot penetrate the scanned object due to the lack of energy intensity, which will lead to noise and artifacts in the reconstructed CT images^6^ and may confuse the identification and analysis of diseases, damaging the diagnostic performance. This leads to the need to remove noise from LDCT.

Various denoising algorithms have been proposed to obtain high‐quality denoised images. The traditional algorithms are mainly divided into three categories: (a) sinogram domain filtration,^7^, ^8^, ^9^ (b) iterative reconstruction (IR),^10^, ^11^, ^12^, ^13^, ^14^, ^15^, ^16^, ^17^, ^18^ and (c) post‐processing of reconstructed image.^19^, ^20^, ^21^, ^22^ IR can significantly improve the quality of low‐dose CT reconstruction images, but it is time‐consuming and complex in practical applications, which is an unavoidable problem. In contrast, sinogram domain denoising and image processing after reconstruction are faster, but they need appropriate models, which may be difficult to obtain, and the problems of resolution loss and edge blur often occur.

With the recent rapid development of deep neural network,^23^, ^24^, ^25^, ^26^, ^27^ the combination of tomography and deep learning or machine learning can not only realize image analysis but also image reconstruction, which provides a new research direction for LDCT denoising. Due to the powerful feature extraction ability of the convolutional neural network (CNN),^28^ it is increasingly applied to image denoising. The methods based on deep learning mainly use advanced DL technology to optimize the structure of the neural network to improve the analysis algorithm. Han et al.^29^ combined with the U‐net model, proposed a new depth residual learning method for sparse view CT reconstruction. Jin et al.^30^ demonstrated the method of low‐field CT reconstruction using filtered back projection (FBP) and CNN combined with residual learning and U‐net architecture. Kang et al.^31^ proposed a deep convolution neural network (CNN) applied to wavelet transform coefficients of low‐dose CT images to suppress specific noise in LDCT. Content‐noise complementary learning (CNCL)^32^ strategy is proposed, which uses two deep learning predictors to learn the respective content and noise of image datasets.

The deep learning method includes two main parts, the neural network model structure and the loss function used to measure the error between the reconstruction result and the target. In the training process, the network model is updated by the backpropagation of the loss function until the error is minimized. In other words, the loss function will directly affect the performance of the model. Although the existing deep learning methods have made various innovations in the model structure and have achieved certain results, they usually use the loss per pixel of the generated image and the target image as the training target, MAE^33^ or MSE,^34^ which leads to two problems. First of all, per‐pixel loss ignores the difference in the difficulty of denoising in each region of the LDCT. The neural network model may ignore the regions that are too difficult to deal with to obtain higher average visual quality output.^35^ In addition, per‐pixel loss makes the reconstructed image lose a lot of texture information which is crucial to human beings. The texture is in the high‐frequency information, but the pixel information is the low‐dimensional feature of the image. So, per‐pixel loss will cause the generated image to become smooth.^36^

To solve the above problems, we propose a hybrid loss function in this study. This hybrid loss function consists of two parts, namely WPLoss and HFLoss. The noise distribution in CT images is not uniform, and experiments show that the noise‐denoising difficulty is uneven in different regions, for example, the noise is more likely to remain in texture‐complex regions, which may be the diagnostic regions of interest to doctors. Therefore, we use WPLoss to perform adaptive noise reduction in different regions of the LDCT image to avoid uneven denoising difficulty in different regions of the image. First, the generated images and the target images are uniformly divided into some corresponding non‐overlapping patches, and then the MAE of each patch is calculated. the larger the patch loss is, the worse the generation effect is, so more training is needed. Therefore, it is necessary to assign a larger weighting factor. Meanwhile, the weights of patches with small losses are weakened to avoid forgetting the trained information due to over‐updating. HFloss is used to measure the difference between the high‐frequency part of the generated image and the target image. The high‐frequency part is extracted by Fourier transform and a high‐pass filter.

In this study, we propose a new hybrid loss function, which can improve the denoising effect of existing denoising models by verifying several models, RED‐CNN,^37^ pix2pix,^38^ SPARNet,^39^ and ldct_nonlocal.^40^ We proved this on the AAPM‐Mayo Low‐Dose CT Grand Challenge dataset and a simulation dataset of 34 patients from The Cancer Imaging Archive (TCIA). By using the proposed hybrid loss function, the LDCT images can be better used to obtain high‐quality images after noise reduction, thus achieving the goal of obtaining accurate diagnostic information at low doses and greatly reducing the risk of radiation to patients.

## 2 METHODS

### 2.1 Noise reduction model

**FIGURE 1:**
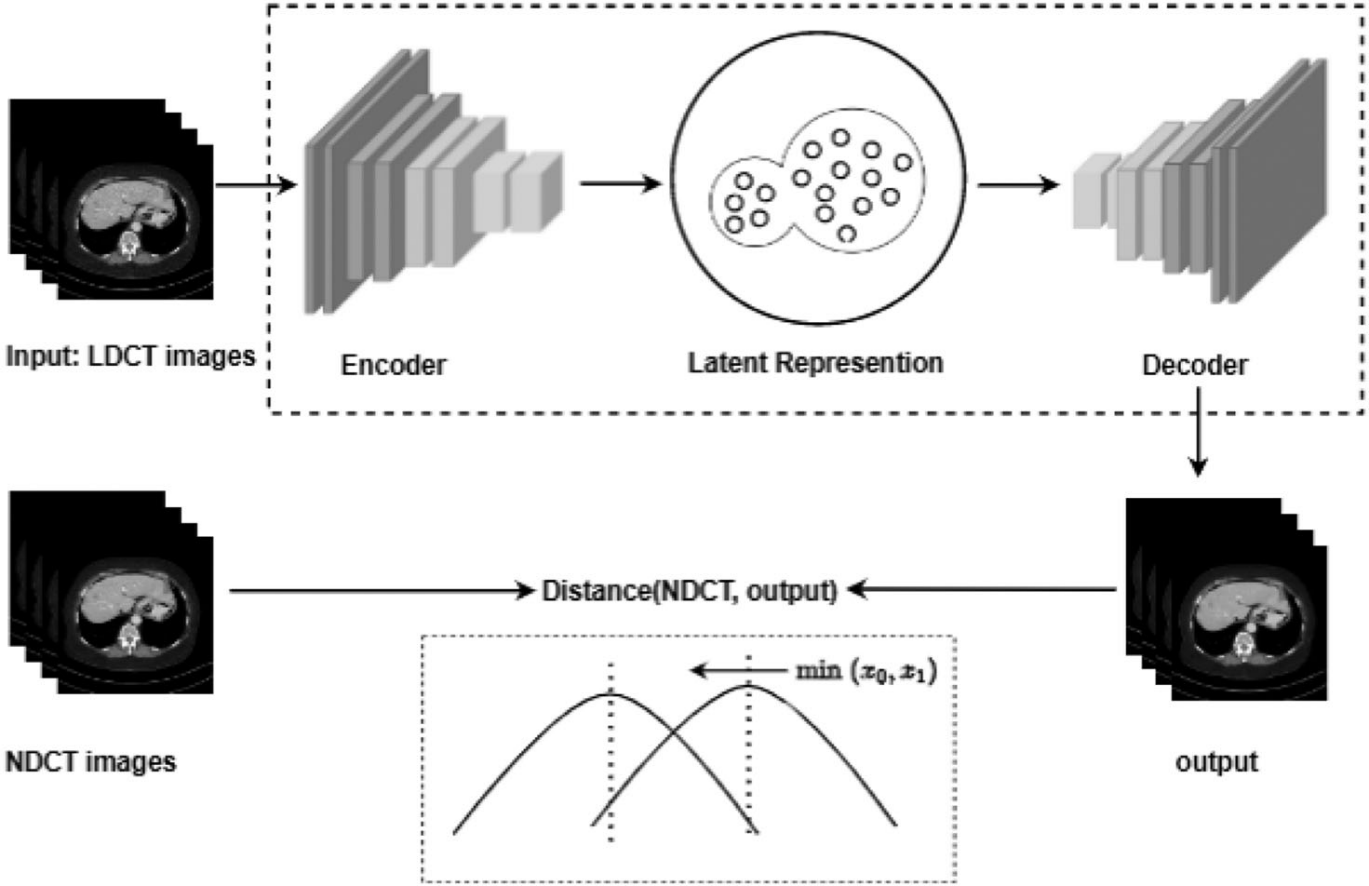
Noise Reduction Model. The model establishes a mapping from input to output. We use LDCT as input, and the goal is to find a model to make the output closest to the corresponding NDCT which means minimizing the *Distance (NDCT,output)*.

As shown in Figure 1, our workflow starts with the images reconstructed by scanning slices, and the denoising problem is limited to the image domain.^41^ Since the method based on deep learning is independent of the statistical distribution of image noise, the LDCT denoising problem can be simplified to the following problem. Suppose that an NDCT image $y\in R^{w\times h\;}$is transformed into an LDCT image $x\in R^{w\times h}$ after adding quantum noise, where w and h mean the length and width of the 2D CT image. Then, their relationship can be expressed as:

(1) $$x=\sigma \left(y\right)$$

where $\sigma :R^{w\times h}\to R^{w\times h}$ denotes the mapping from NDCT to LDCT. A sufficiently complex deep neural network can approximate any function, so by using a series of paired LDCT‐NDCT images (x,y) to train a CNN model ∅ closest to $\sigma^{-1}$ with parameters θ, we can make $\emptyset_{\theta }\left(x\right)$ as close to *y* as possible.

Then, the noise reduction problem can be considered equivalent to finding θ to minimize the Euclidean distance between $\emptyset_{\theta }\left(x\right)$ and *y* after noise reduction:

(2) $$\underset{\theta }{\mathrm{argmin}}E\left({\left|\left|\emptyset_{\theta }\left(x\right)-y\right|\right|}^{2}\right)$$

The backpropagation formula during model updating is expressed as:

(3) $$\theta_{t+1}=\theta_{t}-lr\cdot \frac{dL\left(\theta_{t}\right)}{d\theta_{t}}$$

where $\theta_{t}$ show the parameters of the model at the *t*‐th iteration, $\frac{dL(\theta_{t})}{d\theta_{t}}$ are the gradients corresponding to the model parameters $\theta_{t}$, and lr. is the learning rate.

### 2.2 Hybrid loss function

The hybrid loss function proposed in this paper is expressed mathematically as follows:

(4) $$L_{hybrid}\left(x,y\right)=\alpha_{1}L_{WP}\left(x,y\right)+\alpha_{2}L_{HF}\left(x,y\right)$$

where $L_{WP}\left(x,y\right)$ and $L_{WP}\left(x,y\right)$ denote WPLoss and HFloss which are calculated from Equation (6) and Equation (10) respectively, and α_1_, α_2_ are their weighting coefficients.

#### 2.2.1 WPLoss

Considering that different regions of the image contain different amounts of information and have different denoising difficulties, we fine‐tune the backpropagation process from a new perspective. We propose a new loss function to adjust the loss weight of different regions of the image. In the part with greater difficulty in denoising, there is a large gap between the generated image and the target image during training, so we give a larger weight to improve the model's attention to this area. To achieve this, the generated image and corresponding NDCT image first are divided into N non‐overlapping patch pairs and assume that the loss weight of the i_th pair is a constant $w_{i}$. We define $w_{i}$ as calculated as follows:

(5) $$w_{i}=\left|\frac{\emptyset {\left(x\right)}_{i}-y_{i}}{I}\right|$$

where $\left(\emptyset {\left(x\right)}_{i},y_{i}\right)$ denotes the i_th patches of generated image and NDCT, and *I* is a value in the set $\{|\emptyset {\left(x\right)}_{i}-y_{i}|\},\;1\leq i\leq N$, which is set as the median number in our study. It should be noted that to prevent model instability caused by excessive local loss weight, we limit *w*, for example [0.25,4].

Therefore, WPLoss we proposed is defined as follows:

(6) $$\begin{array}{c} L_{WP}\;\left(x,y\right)=\sum_{i=1}^{N}w_{i}(|\phi {\left(x\right)}_{i}-y_{i}\;\left|\right)\; \end{array}$$

Then, we can replace Equation (3) with the following formula:

(7) $$\theta_{t+1}=\theta_{t}-lr\cdot \frac{dL\left(\theta_{t}\right)}{d\theta_{t}}⊙w$$

In CT images, different areas contain different amounts of information. In experiments, it can be found that the denoising ability of the denoising model is too weak in some places, and may be too strong in others, and the more complex the texture is, the more difficult it is to denoise, but this is often the location of the key pathological information. Here, by adjusting the weight coefficient of the local MAE of the image, we make the model pay more attention to the areas with complex textures, which requires almost no additional calculation work.

#### 2.2.2 HFLoss

Noise reduction models generally add per‐pixel loss to try to minimize the pixel error between the denoised image and the full dose image. Although it makes the generated image have a high PSNR, it may produce blurred images and lead to distortion and texture distortion. The high‐frequency information of the image contains more edge and texture information than the low‐frequency information, so we propose a loss function named HFLoss based on the high‐frequency information.

The discrete Fourier transform^42^ of a two‐dimensional image with length *m* and width *n* is defined as:

(8) $$F\left(u,v\right)=\sum_{x=0}^{m-1}\sum_{y=0}^{n-1}f\left(x,y\right)e^{-2i\pi \left(\frac{ux}{m}+\frac{vy}{n}\right)}$$

The corresponding inverse Fourier transform is expressed as follows:

(9) $$\begin{array}{c} f\;\left(x,y\right)=\frac{1}{mn}\;\sum_{u\;=\;0}^{m-1}\sum_{v\;=\;0}^{n-1}\begin{array}{c} F\left(u,v\right)e^{2i\pi \left(\frac{ux}{m}+\frac{vy}{n}\right)}\\ \; \end{array} \end{array}$$

Through Fourier transformation, we complete the transformation of the image from the spatial domain to the frequency domain. Then we use the high‐frequency filter to keep only the high‐frequency part. Finally, through the inverse Fourier transform, we get the corresponding result of the high‐frequency part of the image, which we think is the edge and texture of the image.

Therefore, the loss function HPLoss we define is calculated as follows:

(10) $$L_{HF}\left(x,y\right)=E\left[\left|f\left(G\left(F\left(\emptyset \left(x\right)\right)\right)\right)-f\left(G\left(F\left(y\right)\right)\right)\right|\right]$$

where *G* denotes the high pass filter.

HFLoss extracts the high‐frequency information of the generated image and the target image and calculates its distance in the data domain to improve the ability of the model to generate detailed textures.

## 3 EXPERIMENTS AND RESULTS

In this section, we first describe the dataset preparation, implementation details, and important parameter settings in our experiment. Then, we conducted qualitative and quantitative experiments to compare the effect of noise reduction before and after adding the hybrid loss to multiple models, and we proved the role of each loss through ablation experiments. Datasets include Mayo datasets and simulation datasets produced using public NDCT datasets. The quantitative evaluation indicators are PSNR and SSIM.

### 3.1 Data source

#### 3.1.1 Mayo data

To verify the effectiveness of the proposed loss function in clinical tasks, we used a real clinical dataset authorized by Mayo Clinic for the “2016‐NIH‐AAPM‐Mayo Clinic low dose CT challenge^43^, ^44^” to train and test. The dataset is made up of 2378 3 mm thickness NDCT images and the corresponding LDCT images of size 512 × 512 from 10 patients. The NDCTs are produced by setting the tube potential to 120 kv and quality effective mAs to 200 mAs. And the Poisson noise is inserted into the NDCTs to obtain the LDCTs corresponding to a noise level of 25% of the full dose. To validate the effect of the proposed loss, we divide the Mayo dataset into training, testing datasets according to 9:1 and training, testing, validation datasets according to 6:2:2 by patient’ number, respectively.

#### 3.1.2 Simulated data

In the simulation dataset, we used 4000 normal doses of CT images of 34 patients downloaded from TCIA^45^ as the NDCT. All images are 512×512 pixels, and 3600 images are used for training, while the rest are used as the testing dataset. The corresponding LDCT dataset is obtained by converting NDCT images to the projection domain by ray transform^46^, ^47^ and adding Poisson noise.^48^ The Poisson noise model can be expressed as:

(11) $$\bar{p_{i}}=K\cdot Poisson\left(N_{0}\cdot e^{p_{i}}\right)$$

Where $p_{i}$ denotes measurement along the i_th path of X‐ray, *K* represents the conversion gain from X‐ray photon to electron, and *N*_0_ is the initial incident intensity of X‐ray used to constrain the intensity of noise. Relative to the Mayo dataset, the TCIA dataset contains more body parts. Moreover, in the simulation dataset, we can freely control the noise level.

### 3.2 Parameter setting

In the experiment, images adjusted to 224×224 are used uniformly, and random clipping is used for data enhancement. In the training, ADAM optimizer with hyperparameters $\alpha =1\times 10^{-5}$, $\beta_{1}=0.5$, $\beta_{2}=0.9$ is used to update the network parameters of all models. In addition, the initial learning rate is set to 10^−4^, and the learning rate is updated in such a way that it decreases by half every 50 epochs. The total training epoch number is set to 400. All convolution and deconvolution kernels are initialized with random Gaussian distributions with zero mean and a standard deviation of 0.01. In the training and testing, the CT images are normalized to [−1, 1] based on the pixel mean and standard deviation. We use peak signal‐to‐noise ratio (PSNR) and structural similarity index (SSIM) as quantitative evaluation indexes to quantitatively evaluate the image quality.

All experiments were carried out on a PC (NVIDIA GeForce RTX 2080Ti GPU) using the PyTorch framework. The parameters of the models used in our experiment are set according to the original papers.

### 3.3 Experimental results

#### 3.3.1 Mayo dataset

In Mayo dataset, the data of nine patients are first selected as the training dataset and the data of another patient as the testing dataset. Table 1 shows the average measurements of all images in the testing dataset. The proposed hybrid loss function as Equation (4) can significantly improve the denoising effect of all models.

**TABLE 1:** Quantitative results of each model with different loss functions for the images in the testing dataset.

Model		RED‐CNN	pix2pix	SPARNet	ldct‐nonlocal
orginal	PSNR	33.8808	30.6886	31.2256	33.2495
	SSIM	0.9327	0.9079	0.8990	0.9295
$+L_{WP}$	PSNR	**33.9251**	32.2338	32.5219	33.4401
	SSIM	0.9028	0.9205	0.9165	0.9303
$+L_{HF}$	PSNR	33.9137	32.1948	31.4470	33.3338
	SSIM	0.9328	0.91722	0.9180	0.9292
$+L_{hybrid}$	PSNR	33.9154	**32.3211**	**32.5415**	**33.4922**
	SSIM	**0.9330**	**0.9207**	**0.91698**	**0.93047**

**FIGURE 2:**
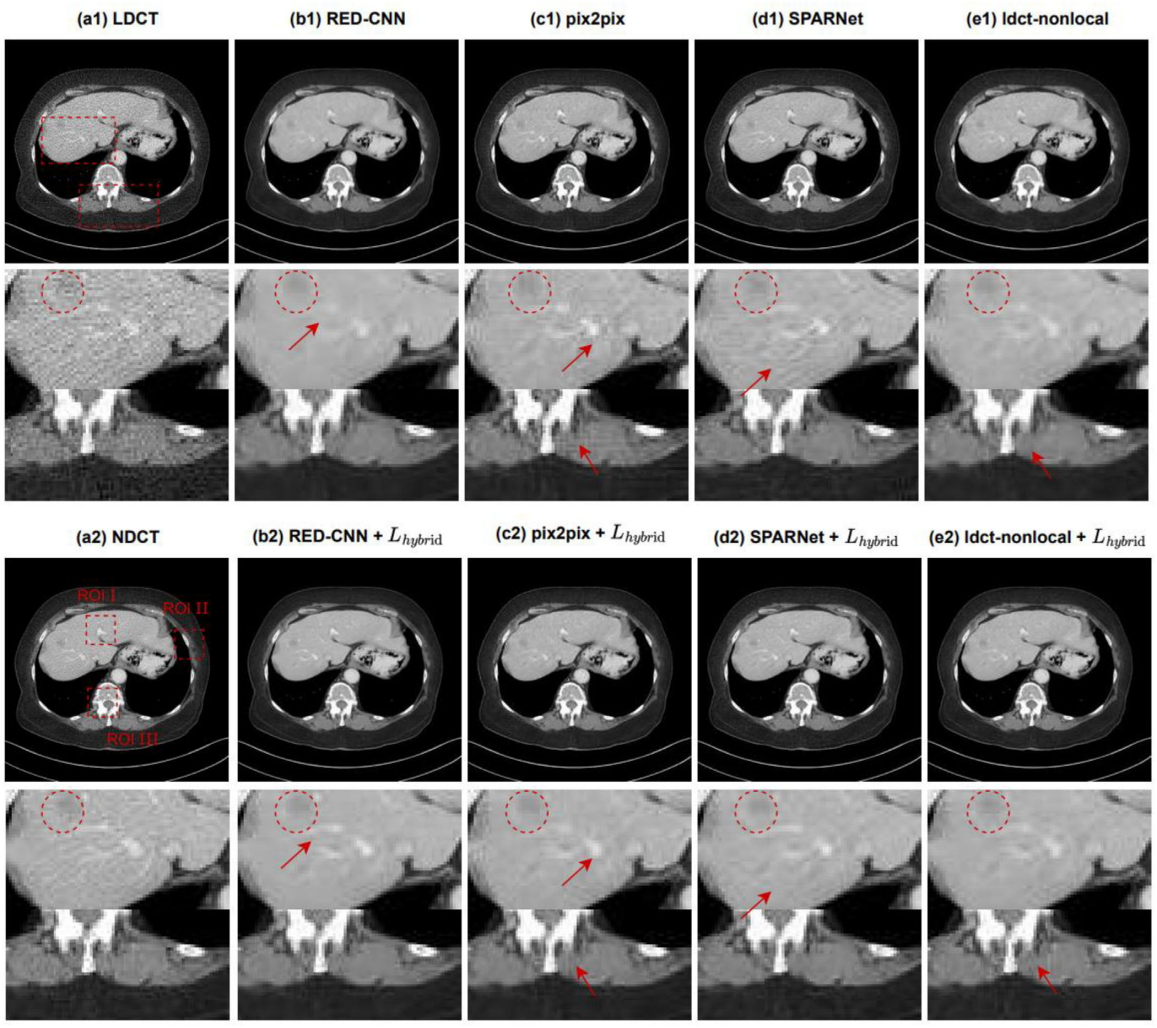
Impact of the hybrid loss function on each model for the abdominal slice in the testing dataset. The images from (a1) to (e1) are LDCT, and the denoising results of RED‐CNN, pix2pix, SPARNet, and ldct‐nonlocal before adding the hybrid loss function, respectively. The two red rectangles in LDCT specify the regions of interest (ROI) and the amplification results are shown in the second row. The images from (a2) to (e2) are the NDCT and the denoising results of each model after adding hybrid loss respectively, and the fourth row is the enlarged images of the corresponding ROI. The arrows indicate the areas where the visual effect are observed. The CT display window is [−160 240] HU. In contrast, after adding the hybrid loss function, every model retains more details and reduces artifacts and blur.

We selected one representative abdominal CT slice in the clinical testing dataset to demonstrate the impact of the hybrid loss function on each model. Figure 2 shows the denoising results of each model before and after adding the hybrid loss function. It can be seen that there is strong quantum noise in LDCT, especially near structural parts (such as bones) with high attenuation coefficient. All models show the denoising effect to varying degrees. However, in the first row, RED‐CNN leads to over‐smoothness and loses a large amount of texture information because it only uses the average per pixel loss as its loss function. In contrast, pix2pix and SPARNet better retain the structural features, but there is still a lot of noise and artifact residue. Though ldct‐nonlocal eliminates most of the noise and artifact and has good structural fidelity, there is still some blur near the bone. The third row shows the denoising results of all models after adding the hybrid loss function. Compared with the denoising results in the first row of the original models, we can find that after adding the hybrid loss function we proposed, all models show a better noise reduction effect and retains more details.

The second and fourth rows in Figure 2 further show the enlarged images of the regions of interest (ROI) in LDCT. By observing the arrow marks, it is obvious that RED‐CNN smoothes some important structures. Pix2pix, SPARNet, and ldct‐nonlocal can retain these details to varying degrees without blurring, but blocky artifacts appear locally, such as near bones. For the low contrast liver lesions highlighted in the red circle, after adding the loss function, all models reduce artifacts and better preserve the edges, improving the detectability of the lesions.

For quantitative evaluation, we select three ROIs as shown in the red dotted boxes of the NDCT image in Figure 2 to calculate PSNR and SSIM respectively. The results are shown in Table 2. It can be found that the quantitative evaluation results are consistent with the visual inspection, and all the ROIs after adding the hybrid loss have higher PSNR/SSIM.

**TABLE 2:** Performance comparison of pix2pix before and after adding hybrid loss over the ROIs marked in Figure 2 in terms of the selected metrics.

	ROI 1		ROI 2		ROI 3	
Model	PSNR	SSIM	PSNR	SSIM	PSNR	SSIM
pix2pix	28.4393	0.7031	29.6335	0.8871	24.9408	0.9001
pix2pix$+L_{hybrid}$	29.0973	0.7144	30.6428	0.8988	26.4292	0.9103

**FIGURE 3:**
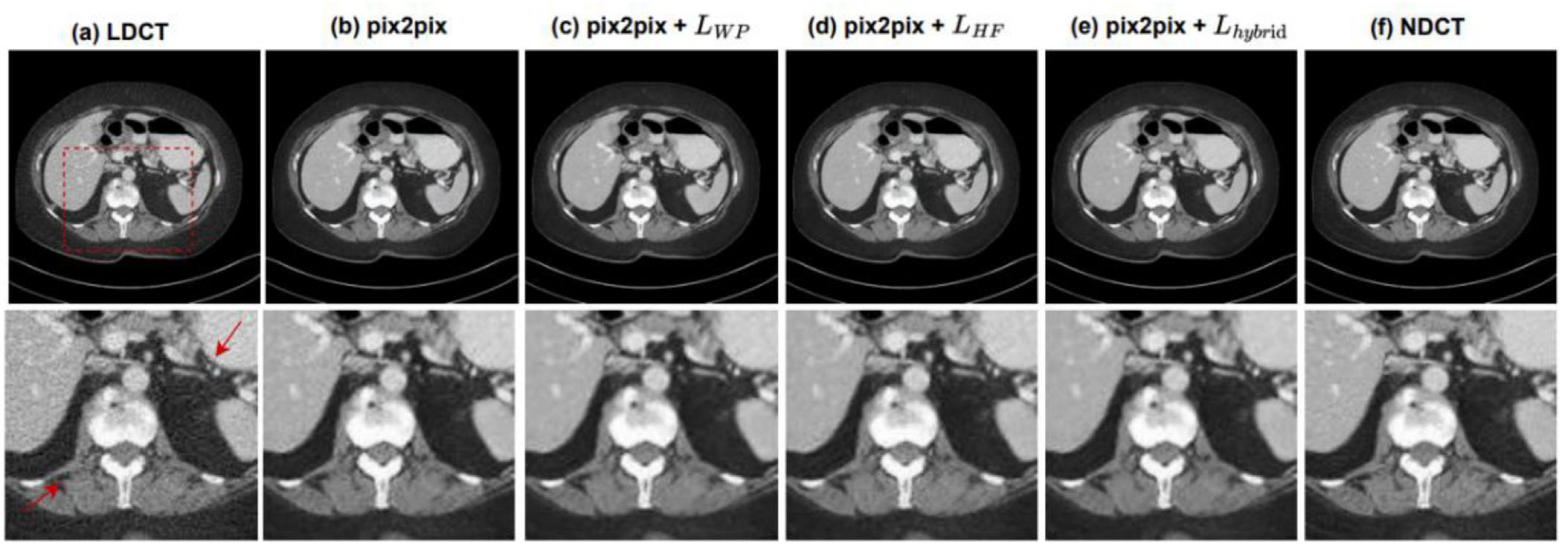
Denoising performance comparison of pix2pix with different loss functions for the abdominal slice in the testing dataset. (a)LDCT, (b)pix2pix with original loss function, (c)adding WPLoss, (d)adding HFLoss, and (e)adding hybrid loss function denoised results, (f)NDCT. The CT display window for the images is [−160 240] HU. The results show that both WPLoss and HFLoss can improve the denoising effect of pix2pix, and when using the hybrid loss, the generated image is the closest to NDCT.

In order to verify the effect of each part of the proposed hybrid loss, we used another abdominal CT slice from the same patient to conduct ablation experiments on pix2pix. Figure 3 shows the noise reduction results of pix2pix with different loss functions. And the second row is the enlarged images of the image content indicated by the red rectangle. By observing the partial images indicated by the red arrows, it can be found that the original pix2pix encountered serious blocky artifacts and blurred near the bone. Although the pix2pix after adding WPLoss reduces artifacts in the image, but there is still the problem of missing texture. In contrast, pix2pix has the smallest difference with NDCT after adding the hybrid loss and retains most of the details.

Table 3 lists the quantitative results from the whole abdominal slice in Figure 3. We can observe that PSNR and SSIM are both improved after adding the hybrid loss we proposed.

**TABLE 3:** Quantitative results associated with pix2pix using different loss functions for the abdominal slice.

Model	LDCT	pix2pix	pix2pix$+L_{WP}$	pix2pix$+L_{HF}$	pix2pix $L_{hybrid}$
PSNR	29.0815	31.3946	32.0056	31.5982	32.0280
SSIM	0.8531	0.9089	0.9120	0.9096	0.9137

Considering that just using the data of 1 patient as the testing dataset can't effectively demonstrate the effect of the proposed loss, we further divided the dataset according to 6:2:2. Besides, to analyze the combined effect on validation dataset, we performed an analysis of variance on the quantitative metrics. The results are shown in Table 4.

**TABLE 4:** Quantitative and analysis of variance r results of each model with different loss functions for the images in the Mayo testing dataset.

Model		RED‐CNN	pix2pix	SPARNet	ldct‐nonlocal
orginal	PSNR	34.4515 ± 3.56	32.1546 ± 4.35	32.0216 ± 3.44	33.7485 ± 3.67
	SSIM	0.9237 ± 0.012	0.9058 ± 0.023	0.9010 ± 0.018	0.9304 ± 0.014
$+L_{WP}$	PSNR	34.6152 ± 3.63	32.4589 ± 4.29	32.6731 ± 3.23	34.2612 ± 3.46
	SSIM	0.9259 ± 0.010	0.9114 ± 0.029	0.9142 ± 0.017	0.9312 ± 0.019

**FIGURE 4:**
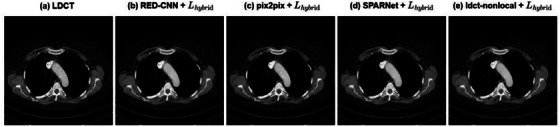
Denoising effect of each model using hybrid loss function on LDCT image of the external testing dataset. The images from (a) to (e) are LDCT image, and the denoising results of RED‐CNN, pix2pix, SPARNet, and ldct‐nonlocal using hybrid loss function, respectively. The CT display window is [−160 240] HU. It can be seen that each model shows a good denoising effect.

To compensate for the lack of data volume in the Mayo dataset, we used an external testing dataset of 20 patients with only LDCT data provided by the Mayo Clinic for further validation. We selected a representative CT image for demonstration, and the results are shown in Figure 4. The proposed hybrid loss in this paper obtained good denoising results on all four models.

#### 3.3.2 Simulation dataset

Table 5 shows the quantitative results of the simulation testing dataset on different models. The results show that our proposed hybrid loss and each part of the loss have improved in quantitative indicators on each model, which provides quantitative evaluation support for visual observation.

**TABLE 5:** Quantitative results of each model with different loss functions for the images in the simulation testing dataset.

Model		RED‐CNN	pix2pix	SPARNet	ldct‐nonlocal
orginal	PSNR	37.2063	33.1902	33.8622	36.5580
	SSIM	0.9800	0.9671	0.9669	0.9779
$+L_{WP}$	PSNR	37.2019	33.9017	33.9996	**36.7662**
	SSIM	0.9800	0.9695	0.9677	0.9791
$+L_{HF}$	PSNR	37.2067	33.2652	34.1113	36.6614
	SSIM	0.9799	0.96722	0.9682	0.9786
$L_{hybrid}$	PSNR	**37.2080**	**33.9278**	**34.1361**	36.7368
	SSIM	**0.9810**	**0.97008**	**0.9683**	**0.9791**

**FIGURE 5:**
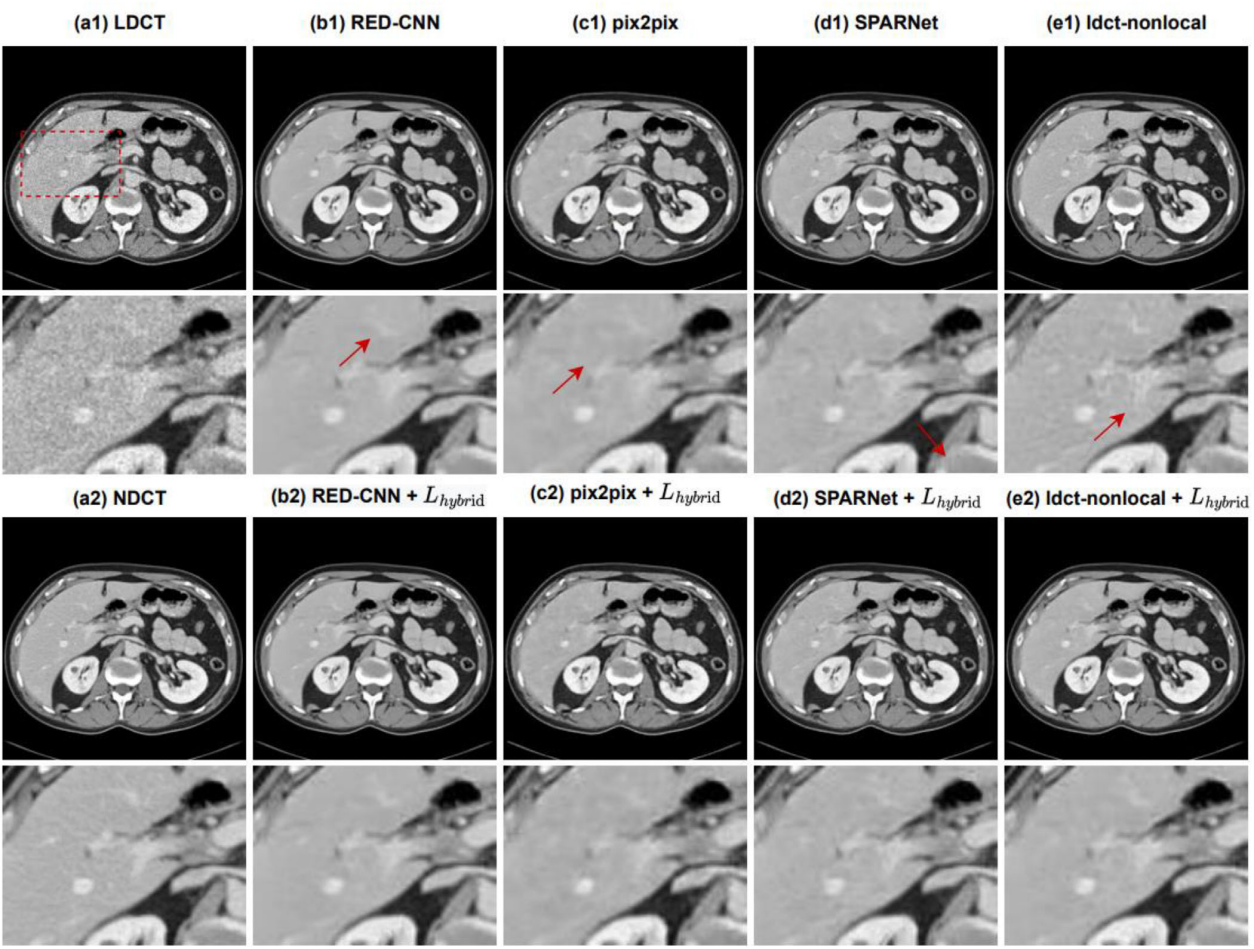
Impact of the hybrid loss function on each model for the abdominal slice in simulation testing dataset. The images from (a1) to (e1) are LDCT, and the denoising results of RED‐CNN, pix2pix, SPARNet, and ldct‐nonlocal before adding hybrid loss function, respectively. The images from (a2) to (e2) are the NDCT and the denoising results of each model after adding hybrid loss respectively. The red rectangle in LDCT specifies the ROI and the amplification results are shown in the second and the fourth row. The CT display window is [−160 240] HU. In contrast, after adding the hybrid loss function, every model retains more details and reduces artifacts and blur.

Figure 5 shows the influence of the hybrid loss function on the denoising effect of each model. The second and fourth rows are the zoomed images over the ROI. By observing the parts marked by the red arrows, it is obvious that all models have improved in noise suppression and structure maintenance after adding the hybrid loss. On the contrary, in the original models, RED‐CNN and pix2pix smoothed some details. Although SPARNet and ldct‐nonloacl retained more details, blocky artifacts appeared locally.

**FIGURE 6:**
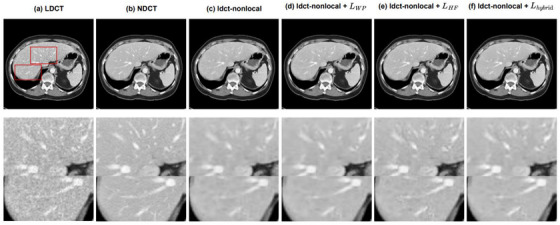
Denoising performance comparison of ldct‐nonlocal with different loss functions. (a)LDCT, (b)NDCT, (c)ldct‐nonlocal, (d) ldct‐nonlocal adding WPLoss, (e) ldct‐nonlocal adding HFLoss, and (f)ldct‐nonlocal adding the hybrid loss denoised results. The display window for the images is [−160 240] HU.

Figure 6 shows the denoising results of another abdominal slice in the testing dataset with different loss functions on ldct‐nonlocal. Obvious blocky artifacts and blurring appear after ldct‐nonlocal denoising. By visually inspecting the local magnified images, the denoising result has a clearer appearance after adding WPLoss, but there is still the problem of unclear edges, and adding HFLoss makes up for the problem of blurred edge texture.

To analyze the combined effect of the model on testing dataset, we performed an analysis of variance on the quantitative metrics. The results are shown in Table 6.

**TABLE 6:** Analysis of variance results for quantitative metrics on the testing dataset.

	Model	RED‐CNN	pix2pix	SPARNet	ldct‐nonlocalll
orginal	PSNR	37.2063 ± 3.03	33.1902 ± 3.97	33.8622 ± 3.54	36.5580 ± 3.14
	SSIM	0.9800 ± 0.010	0.9671 ± 0.033	0.9669 ± 0.024	0.9779 ± 0.011
$+L_{WP}$	PSNR	37.2080 ± 2.95	33.9278 ± 3.53	34.1361 ± 3.25	36.7368 ± 3.06
	SSIM	0.9810 ± 0.013	0.9701 ± 0.029	0.9683 ± 0.020	0.9791 ± 0.017

## 4 DISCUSSION

As a multi‐level imaging technology, CT can provide high‐resolution, high‐quality three‐dimensional images. In practical applications, CT is commonly used to diagnose a variety of diseases, such as lung diseases, neurological diseases, and bone diseases, which can provide more accurate diagnostic information and help doctors better develop treatment plans. However, the X‐rays used in CT produce radiation, and long‐term exposure to radiation may increase the risk of developing cancer, which greatly limits the use of CT in clinical applications. There are means to reduce the radiation dose and obtain low‐dose CT results, thus reducing the associated risks. Low‐dose CT images have important medical significance and diagnostic value, such as liver, pancreas and breast examinations, and can promote disease prevention and early diagnosis, early detection of lesions, and improved treatment outcomes. However, low‐dose CT has a large amount of quantum noise, which affects doctors' diagnosis of the condition, thus reducing the diagnostic value of low‐dose CT. With the explosion of deep learning in recent years, various deep neural network models have been developed to obtain high‐quality images using low‐dose CT. However, most of them optimize the structure of neural networks to achieve better denoising. Appropriate loss functions are important for neural network models, but most of them usually use per‐pixel loss such as MAE and MSE. however, per‐pixel loss ignores the difficulty of denoising CT images without region and the generated images often suffer from texture detail loss.

Various loss functions have been proposed by many national and international researchers for improving the denoising performance of the models. Perceptual loss^49^ uses neural networks to extract high‐level features of images, which effectively promotes the approximation of denoising results and real images in terms of deep information. However, perceptual losses may have some problems when processing images with higher complexity, for example, more complex textures, shapes, etc. may be ignored, in contrast, our proposed HPFLoss uses converting images from the image domain to the frequency domain through Fourier transformations and filters out low‐frequency information, effectively retaining texture details in high‐frequency information. Jiang^50^ et al. proposed focal frequency loss by reducing the simple frequency components to focus adaptively on the difficult‐to‐synthesize frequency components, effectively facilitating the generation of texture details by the model. However, the frequency domain information consists of frequency components containing complex relationships, and low‐frequency information can interfere with the generation of high‐frequency information. Therefore, the high‐pass filter is used in the HPFLoss proposed in this paper to filter low‐frequency information and focus on the generation of high‐frequency information, while WPLoss is used to calculate the image pixel loss, which ensures the retention of low‐frequency information.

The advantages of our proposed hybrid loss are its interpretability and simplicity. WPLoss does not require any additional computational operations and only requires reassigning the loss weights for each region in the image. However, it is important to note that the weight needs an appropriate threshold to avoid model shocks caused by too large weights. The difference between the high‐frequency part of the image generated by the HFLoss calculation and the corresponding NDCT image penalizes the model when the texture details are not well generated. We combine these two complementary loss functions to form a new hybrid loss function. Since these two loss functions have different roles, we need to balance their effects in practical applications. We find the optimal parameters through extensive experiments to make them widely adaptable.

One of the main drawbacks of our study is the lack of a dataset. The clinical dataset contains data from 10 patients, 9 of which were used for training. Due to the small number, the model may lack generalization ability. However, we believe that with large‐scale internal validation and the random nature of the data, our hybrid loss function can achieve better results. We validated it on several models and demonstrated that it is a good component to add to existing models and can meet clinical requirements. In addition, the dataset is not clear enough with a large number of blurred or low‐quality images. We used manual screening to avoid the impact of this problem. In addition, we are collecting more data.

In summary, we propose a hybrid loss function for LDCT image noise reduction, and we have demonstrated that the loss function can effectively improve the denoising performance of the model.

## AUTHOR CONTRIBUTIONS

Investigation and methodology: Zhenchuan Wang, Minghui Liu, Xuan Cheng. Manuscript writing and revision: Zhenchuan Wang, Jinqi Zhu, Xiaomin Wang. Analysis data and Curation: Haigang Gong, Lifeng Xu. Conceptualization: Ming Liu.

## CONFLICTS OF INTEREST STATEMENT

The authors declare no conflict of interest.

The simulation data that support the findings of this study are available in [TCGA‐LUAD] at [https://nbia.cancerimagingarchive.net/nbia-search/], reference number.^44^

## Data Availability

The Mayo data that support the findings of this study are available in [2016 Low Dose CT Grand Challenge] at [https://doi.org/10.7937/9npb-2637], reference number.^43^
